# Simulation and Experimentation of a Grounding Network Detection Scheme Based on a Low-Frequency Electromagnetic Method

**DOI:** 10.3390/s23167254

**Published:** 2023-08-18

**Authors:** Qingming Duan, Bofeng Zou, Yuxin Song, Yuxiang Liu, Ruipeng Zhang

**Affiliations:** College of Instrumentation & Electrical Engineering, Jilin University, Changchun 130012, China; zoubf21@mails.jlu.edu.cn (B.Z.); yuxins22@mails.jlu.edu.cn (Y.S.); udhndimeosl@sina.com (Y.L.); zhangrp@jlu.edu.cn (R.Z.)

**Keywords:** grounding network, low-frequency electromagnetic method, excitation source, node-to-node conductor

## Abstract

The grounding network is a significant component of substations, and the corrosion of its ground resistance is predominantly detected using the electromagnetic method. However, the application of electromagnetic methods for detecting corrosion within earthing networks has received relatively limited attention in research. Currently, the prevailing method utilizes electromagnetic techniques to identify the breakage points within the given earthing network. In this study, we propose a corrosion detection method for grounding networks based on the low-frequency electromagnetic method, which measures the resistance value between individual nodes of the network. Specifically, an excitation source signal of a predetermined frequency was transmitted to the measurement segment of the grounding network, which facilitated the direct measurement of the strength of the induced magnetic field above the center of the measuring conductor. The recorded electromagnetic data were subsequently uploaded to the host computer for data processing, and the computer interface was constructed based on a LABVIEW design. By leveraging the relationship between the induced electric potential, current strength, excitation source strength, and additional voltage detection devices, the resistance of the conductor under examination could be determined. Furthermore, the proposed method was tested under suitable conditions, and it demonstrated favorable results. Thus, the proposed method can serve as a foundation for developing electromagnetic testing instruments tailored to the investigated grounding network.

## 1. Introduction

The grounding network constitutes a crucial component within electrical systems, including substations [1,2]. Over time, factors such as environmental conditions and prolonged equipment operation can cause corrosion within the grounding network. This corrosion is typically characterized by the thinning or breakage of conductors at specific junctions within the grounding network [3]. Thus, corrosion detection within the grounding networks holds tremendous significance. Consequently, several methods are employed for corrosion detection in grounding networks, including electrochemical, electrical network, multi-information fusion, and electromagnetic detection methods [4,5,6,7]. In particular, the electrochemical method focuses on assessing corrosion within the grounding network by examining the electrochemical characteristic parameters within the grounding conductor and the surrounding soil. These parameters include polarization resistance, breakdown potential, and corrosion current, among others [8,9]. Vernon R. Lawson’s team conducted experiments considering parameters such as grounding resistance and polarization current to estimate the corrosion rate of buried metal. However, this method is susceptible to environmental factors such as temperature and soil moisture [10]. The electrical network method incorporates analog circuit fault determination parameters into grounding network detection [11]. Although this method is labor-intensive and involves complex wiring, it incurs higher labor and material costs, as well as increased data processing time. As the name implies, multiple information fusion methods can simultaneously detect corrosion within the grounding network while integrating and interpreting the degree of corrosion and other parameters obtained through various detection techniques. This approach effectively enhances the accuracy of test results [12]. Nevertheless, the multi-information fusion method currently lacks a robust theoretical framework and practical viability. The electromagnetic method, based on the principle of electromagnetic induction, utilizes the detection of the magnetic field generated by excitation to determine the information and state of the grounded conductor [13]. The low-frequency electromagnetic method falls within the purview of AC excitation electromagnetic techniques that can measure low-frequency magnetic fields between two points excited by a noninduced current from an artificial excitation source. Therefore, the amplitude of the measured current is obtained by measuring the strength of the magnetic field at low frequency [14]. This method does not require the excavation of the soil and can save costs.

The primary objective of this study is to simulate the grounding resistance model of a grounding grid using Comsol and Multisim software. The simulation entails the detection of the magnetic field strength directly above the center of the measured conductor by injecting a specific frequency excitation source into both ends of the conductor. By comparing the calculated current values with those obtained through Multisim, the feasibility of the low-frequency electromagnetic method for corrosion detection in grounding networks can be demonstrated. Subsequently, field experiments were conducted utilizing wires and resistors. The experimental findings substantiated the practical viability of this method.

## 2. Materials and Methods

In this section, we designed a scheme for detecting the resistance in grounding networks, specifically targeting the resistance between nodes. Subsequently, an analysis was conducted using a joint simulation of the multi-physics field simulation software Comsol and the circuit simulation software Multisim. The simulation results validated the feasibility of utilizing the low-frequency electromagnetic method to detect resistance between nodes within the grounding network. Furthermore, practical experiments were conducted in the field, where a “grounding network” was constructed using power resistors. These experimental results verified the feasibility of this approach for detecting corrosion in grounding networks.

### 2.1. Basic Principles of Detection

**Figure 1:**
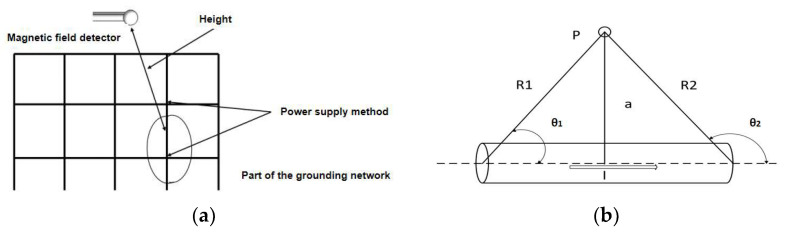
(**a**) Detection scheme and (**b**) detection schematic.

The grounding network comprises multiple interconnected nodes. According to the actual grounding network characteristics, without excavating the soil, we can only access the nodes. To realize more effective detection, we focused on measuring the detection range of the grounding network between the nodes. By adopting this approach, the resistance value of the grounding conductor could be determined based on pairs of nodes as reference points. To determine the resistance value of the grounded conductor, the theoretically simplest approach is to employ the formula R = U/I. While the voltage, U, can be acquired by connecting a voltmeter or voltage detector in parallel to the measurement interval, the primary consideration lies in determining the most convenient method to obtain the current, I. Drawing upon the concept of the low-frequency electromagnetic method and the Biot–Savart law [15], when we inject alternating current at the ends of the conductor under test, the excitation current flowing through the conductor under test can produce a magnetic field in the surrounding. It becomes possible to detect the resulting magnetic field on the proper location. The magnetic field detector was employed for electromagnetic data acquisition at the appropriate location above the conductor under examination. The higher the frequency of the electromagnetic field, the faster it decays, so we chose to use a low-frequency excitation source to provide an additional incentive. Then, we detected low frequency magnetic fields generated by low frequency currents inside the conductor under test. In summary, the strength of the induced magnetic field was utilized to determine the magnitude of the current flowing within the conductor under examination. At this time, the resistance of the conductor under test could be solved. The detection scheme is shown in Figure 1a, and the schematic diagram of the magnetic field generated by the current in the conductor is shown in Figure 1b. The lowest cylinder is the conductor to be tested, P is the magnetic field measurement point, and this is also the location of the magnetic field detector. A is the vertical distance from the measurement point to the conductor under test, and R is the linear distance from the current element in the conductor under test to the detection point P.

The magnitude of the magnetic induction *dB* produced by the current element *IdL* at a point *P* in space is proportional to the magnitude of *IdL*, expressed as

(1) $$dB=\frac{\mu Idl}{4\pi R^{2}}sin\theta$$

where *Idl* represents the current element, *dB* denotes the magnetic induction of the current element at a point *P* in space, *θ* indicates the angle between R and the current element *Idl*, and *μ* denotes the magnetic permeability of the medium, $\mu ={s\mu }_{0}$ where $\mu_{0}=4\pi \times 10^{-7}H/m,$ *s* denotes the relative magnetic permeability, and it is generally taken as 1.

Therefore, the magnetic induction of the entire current-carrying wire at point *P* can be expressed as

(2) $$B=\int_{L}\frac{\mu Idl}{4\pi R^{2}}sin\theta$$

*L* denotes the integration path, i.e., the conductor under test length. 

If the length *L* of the conductor and the vertical distance *a* from the measurement point to the conductor are known, the magnetic field strength at point P can be rewritten as

(3) $$B=\frac{\mu I\left(cos\theta_{1}-cos\theta_{2}\right)}{4\pi a}$$

When the measurement point *P* is located directly above the center of the measurement conductor, ${\cos\theta }_{1}=-{\cos\theta }_{2}$, the magnetic field strength was expressed as

(4) $$B=\frac{\mu I}{2\pi a}cos\theta_{1}$$

Therefore, the current flowing through the measurement interval can be solved for

(5) $$I=\frac{2\pi aB}{\mu cos\theta_{1}}\;$$

Therefore, the strength of the detected current can be solved. In order to best conduct the experiments, fixtures of a specific height are needed here to hold the coil sensors. The resistance of the conductor can be measured by solving for R=U/I.

### 2.2. Simulation Model Design and Parameter Design

**Figure 2:**
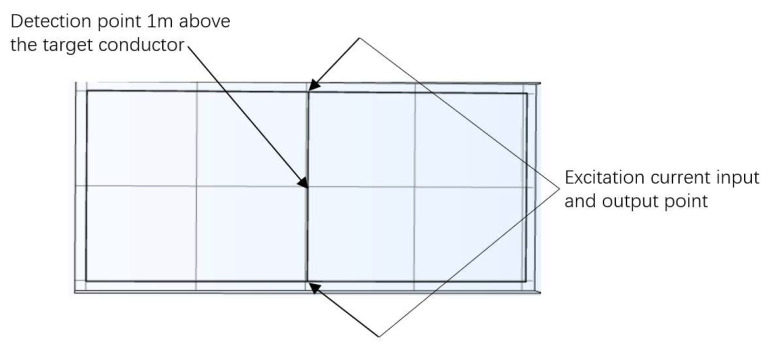
Grounding network simulation model on Comsol.

Comsol has electromagnetic simulation capabilities. We used Comsol to simulate the relationship between the distribution of the current and magnetic field in the grounding network. The scheme we have designed measures the resistance between the nodes. The interference mainly came from the conductor current around the target conductor; the effect of current in other conductors is too small. Therefore, we do not have to think too much about it and we do not need to build an overly complex grounding network model when simulating, just pay attention to the target conductor. We designed the grounding network simulation model in Comsol, as illustrated in Figure 2, where the designed detection range exhibited a “day-shaped” simulation model. The conductor positioned in the middle was selected as the target for testing, and an excitation current of a specific frequency was injected at its two ends. To mitigate interference from industrial frequency and its odd harmonics, as well as the characteristics of low-frequency magnetic fields, a sinusoidal signal with a frequency of 380 Hz was set as the excitation current. For enhanced detection accuracy, the magnetic field was measured directly above the center of the conductor under examination. Generally, grounding networks are not buried deeper than one meter. Therefore, for practicality, and ease of calculation, a detection point situated one meter directly above the conductor was selected. That was at the suitable position of grounding surface.

**Figure 3:**
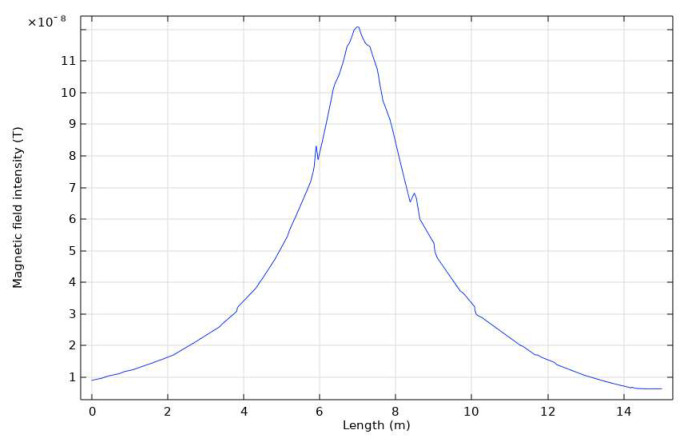
Simulation results of Comsol.

Low-resistivity materials such as copper and galvanized steel are commonly used for grounding network materials. They have a resistivity of around 1.72 × 10^−8^ Ω·m, and the dry soil resistivity is generally in the range of 100~500 Ω·m. Therefore, when we conduct a simulation of a grounding network conductor under test, the conductor’s electrical conductivity is set to 1.72 × 10^−8^ Ω·m, the soil resistivity to 100 Ω·m, and the relative magnetic permeability is 1, the conductor diameter to 40 mm, the conductor length between nodes is 10 m, the location is 1 m below the surface soil, the excitation current is a sinusoidal current, and the effective value of the excitation current to 1 A and the frequency is 380 Hz. The graph of the result without corrosion is shown in Figure 3. The vertical axis is the magnetic field strength, and the horizontal axis is the length coordinate of the detection line. The location with the highest magnetic field strength in the figure is the detection location of the target conductor. We can change the resistivity to simulate the high resistance caused by the appearance of corrosion. Finally, we performed the calculation and recording of the target current using Equation (5).

**Figure 4:**
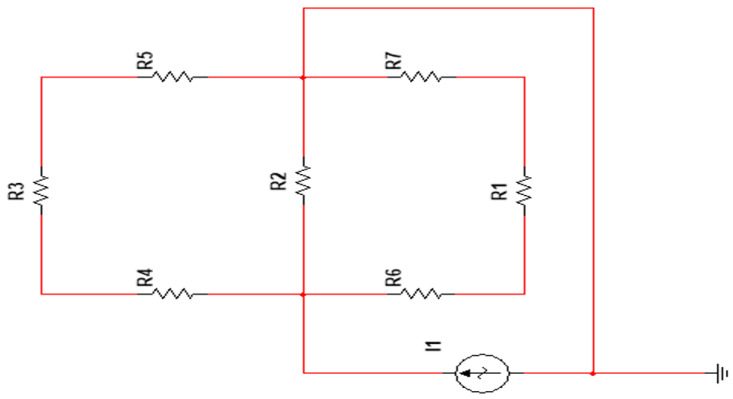
Grounding network simulation model on Multisim.

At the end of the Comsol simulation, we constructed a circuit simulation model of the same scale in the circuit simulation software Multisim. The purpose of this was to get for the specific current value flowing through the target conductor in the most perfect situation. Simulation in Multisim is equivalent to measuring the target current without any interference. Here, we used the idea of the electrical network method of grounding network detection. We equated grounding networks to purely resistive networks. This corresponded to the combination of a resistor and wires [16]. The resistance of R1 to R7 was equal in magnitude to the resistance of the node conductors in Comsol. The excitation current conditions in Multisim are the same as in Comsol. The effect of corrosion was achieved by changing the resistance of the target resistor R2. The Multisim circuit simulation diagram is illustrated in Figure 4.

After completing the Comsol and Multisim simulations, the resulting current strength by Multisim was compared to the existing current strength obtained by converting the magnetic field strength from Comsol. We mainly reached the difference in current for different levels of corrosion. The degree of corrosion means how much greater the resistance value is after corrosion. In China, mild corrosion is becoming bigger by 0 to 1, moderate corrosion is becoming bigger by 1 to 9, and severe corrosion is more extensive than 9 times. We can consider whether to replace the conductor when it is more extensive than 4~6 times. The results are summarized in Table 1.

The results indicated that as the corrosion of the conductor under test increased, the difference between the current values converted by Comsol and the current values measured directly on Multisim increased continually. However, the difference was considerably smaller than the actual current value in the case of less severe corrosion, and the current flowing through the target after the corrosion has become 5 times larger was much smaller than in the case without corrosion. Therefore, the simulation results demonstrated the feasibility of the low-frequency electromagnetic method and the proposed detection scheme for detecting corrosion in the grounding network. And, together with the literature [16,17], leakage currents in dry soils were minimal and need not be considered too much. The grounding grid experimentation had essentially the same effect in dry soil as in dry air. However, ensuring that the coil sensor and the target conductor are experimented with in the same medium is important. And wet environments for the experiment also have a certain degree of danger, so we, as a result of this, recommend that the experiment is conducted in a dry, sunny environment.

### 2.3. Coil Detection Model

**Figure 5:**
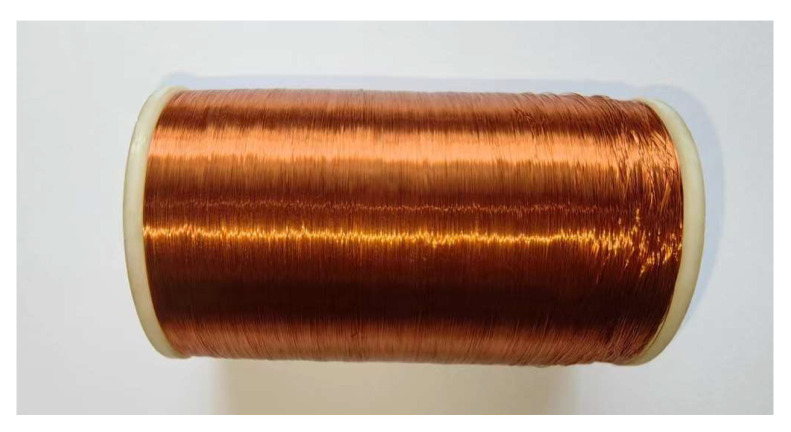
Physical model of the induction coil.

The current can be indirectly obtained by measuring the accompanying magnetic field. Therefore, how to measure the magnetic field strength is the focus here. The coil sensor generates an induced electric potential in the presence of a magnetic field. Therefore, we used a coil sensor to test the magnetic field, which comprised two fundamental components: the coil itself and a post-added operational amplifier. The physical model of the induction coil is shown in Figure 5.

According to Faraday’s law of electromagnetic induction, the induced electric potential generated by the induction coil can be expressed as follows:

(6) $$e\left(t\right)=-n\frac{d\Phi \left(t\right)}{dt}=-nS\frac{dB\left(t\right)}{dt}$$

where *Φ* denotes the induction flux inside coils, *n* denotes the number of turns of the coil, and S represents the effective induction area of the coil.

The induction electromotive force in the frequency domain can be expressed in the following form:

(7) e=−jωnSB

where ω represents the angular frequency of the induced magnetic field.

The above equation shows that the magnetic field is proportional to the induced electric potential, all other things being equal. And the magnetic field is also proportional to the excitation current. Therefore, the current value can be solved from the induced electric potential generated by the coil.

**Figure 6:**
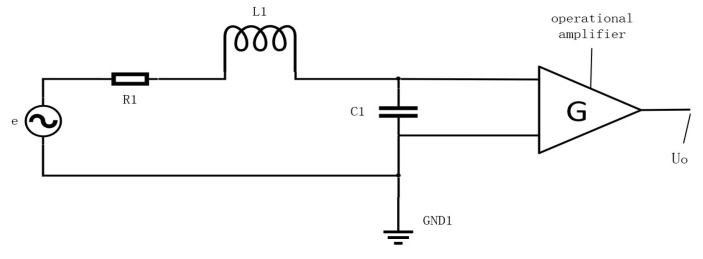
Equivalent circuit model of induction coil sensor.

Since the coil sensor is formed by winding tiny wires wrapped in insulating paint around a cylindrical skeleton. Therefore, the wire’s own resistance $R_{1}$, the distributed capacitance $C_{1}$, and the distributed inductance $L_{1}$ due to the way the circle is wound are all factors. Usually, a capacitor is connected in parallel at the output of the coil. This allows the resonant frequency of the coil to be changed so that the resonant frequency is equal to the magnetic field frequency. Therefore, the coil sensor can be equated to an *RLC* resonant circuit [18]. The equivalent circuit model of the induction coil sensor is illustrated in Figure 6. The output signal is evaluated as follows:

(8) $$U_{O}=\frac{Ge}{1-\omega^{2}C_{1}L_{1}+j\omega R_{1}C_{1}}=-\frac{j\omega NSGB}{1-\omega^{2}C_{1}L_{1}+j\omega R_{1}C_{1}}$$

where $R_{1}$ denotes the resistance of coil, $C_{1}$ denotes the sum of the own capacitance of the coil and the external parallel capacitance value, and $L_{1}\;$ denotes the inductance of coil. Although these coil parameters can be calculated, they might not be accurate. Thus, we need to measure the parameters with the electric Bridge or using other existing measurement tools.

Due to the circuit being special, the induction coil possesses a specific frequency point at which the coil circuit exhibits purely resistive characteristics. This frequency point is referred to as the resonance frequency. At the resonant frequency, the output signal amplitude attains its maximum value. Therefore, the resonant frequency of the coil needs to be made the same as the frequency of the excitation field by means of a parallel capacitor. The resonant frequency of the coil was calculated as follows:

(9) $$f=\frac{1}{2\pi \sqrt{L_{1}C_{1}}}\;$$

where $C_{1}$ denotes the sum of the own capacitance of the coil and the external parallel capacitance value. Equation 9 allows us to select the desired resonant frequency of the induction coil by connecting a capacitor in parallel with the coil after it has been produced. Therefore, when the frequency is known, the capacitance of the specific capacitor to be connected in parallel can be found. Its capacitance value is $C_{1}-C_{l}$.

### 2.4. Field Experiment

**Figure 7:**
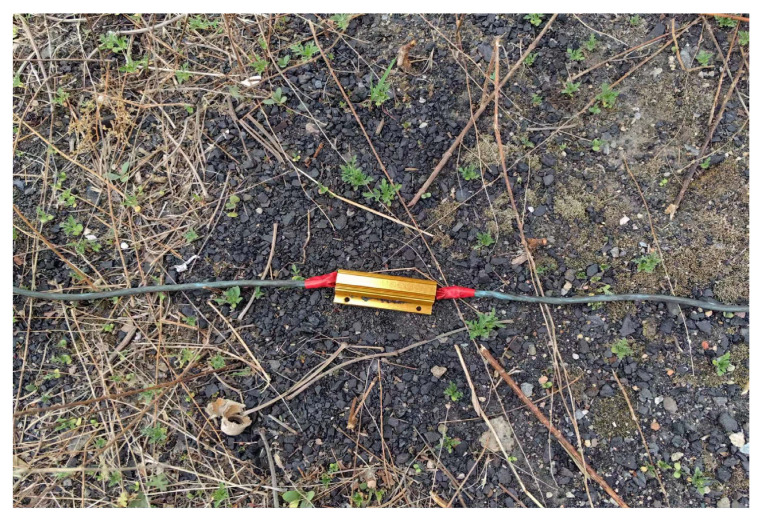
Image of simulated conductor.

In this section, we verified the feasibility of the proposed scheme and method for corrosion detection in a grounding network through practical verification. We set up practical experiments in the field. The resistance between grounding network nodes was simulated by connecting power resistors of different resistance values in series with field wires. The corrosion of the conductor under test in the earthing network was portrayed by varying the resistance of the power resistor. The grounding network grid lines are illustrated in Figure 7.

**Figure 8:**
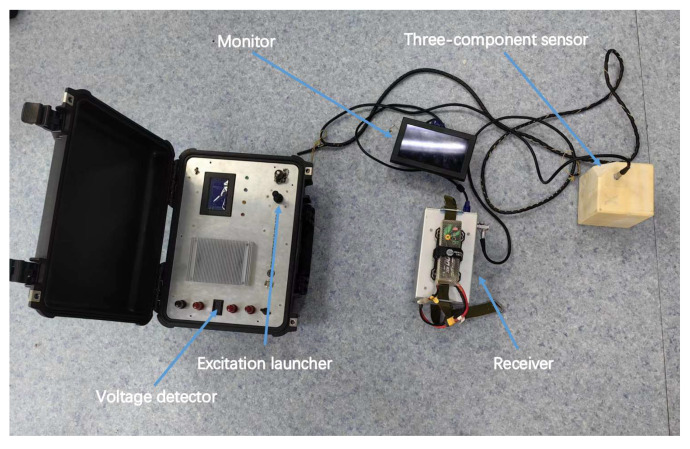
Overall diagram of testing instrument.

According to the proposed program, the applied testing apparatus is depicted in Figure 8. The detection instrument was segmented into two parts: the excitation emission and signal reception. The primary function of the transmitter section was to transmit 380 Hz sinusoidal excitation. The function of the receiver section was to receive the induced magnetic field from the current in the conductor under test between the nodes of the conductor under test and the voltage amplitude. To avoid errors due to sensor shake during detection, we used three-component sensors that could measure the total field strength. Each coil of this three-component sensor was perpendicular to each other, just like a three-dimensional coordinate axis. Consequently, the total field strength directly above the measured conductor could be detected even if the sensor oscillated. Each component contained 8000 coil turns, with an own inductance of approximately 1 H and own resistance of approximately 120 Ω, and the capacitance value after tuning was approximately 0.175 μF. The sensitivity of the sensor was 172 mV/nT@380 Hz.

All detection signals were processed and captured by the acquisition card. In order to facilitate the acquisition, we used the Xinchao USB-2404 integrated capture card for acquisition [19]. The Xinchao USB-2404 offers four analog signal inputs with high accuracy, which is adequate for the required acquisition.

**Figure 9:**
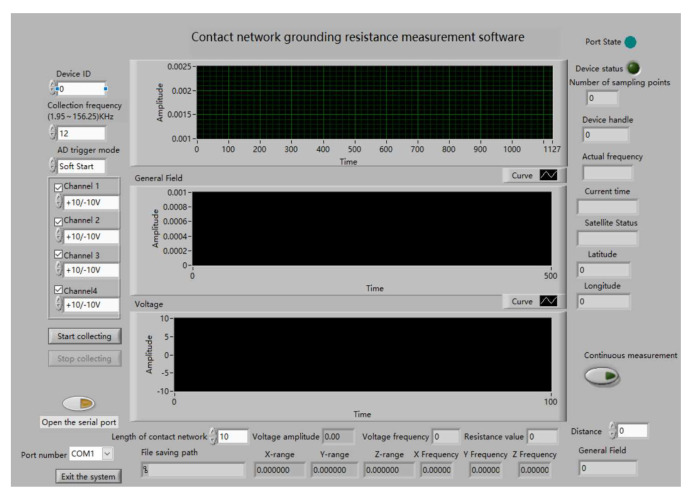
Upper computer software operation interface.

The upper computer is an essential component of the receiver. The role of the upper computer is mainly for the operator to use as an operator interface. LABVIEW edits the upper computer control software. It can control the acquisition status of the acquisition card and the display and storage of test data to meet experimental requirements [20]. The upper computer software operation interface is displayed in Figure 9. We used three channels to collect the induced electromotive force in the X, Y, and Z directions. Finally, a suitable amount of synthesis was conducted to find the total induced electromotive pressure.

**Figure 10:**
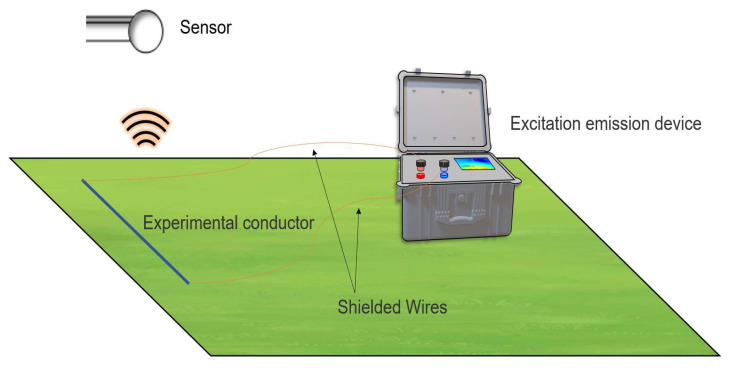
Schematic of measurement wiring.

**Figure 11:**
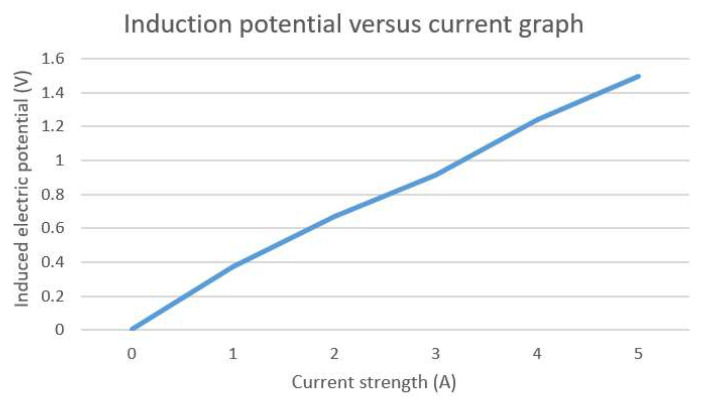
Induction potential versus excitation current.

As the received signal is the signal after both the coil itself and the signal processing, we could not use it directly. To solve this problem, we connected a set of “conductors” to the excitation source and recorded the changes in the induced electric potential directly above it for different excitation currents. The operational layout is depicted in Figure 10. The experimental site was on the more remote outskirts of Changchun, Jilin Province, China. The soil there had been kept dry for 7 consecutive days and was suitable for experimental needs. When conducting experiments, we used the known conditions to determine the approximate range of the excitation current, such that it would not pose a safety hazard or produce a lack of data. The inductive electric potential versus the excitation current is graphically plotted in Figure 11. As long as the induced electromotive force was detected within this range, the corresponding excitation current strength could be obtained.

Although there are magnetic disturbances from currents within conductors between other nodes and from leakage currents in the grounded network, their influence on the target detection current is known to be small by simulation results and references [17]. Therefore, we think using this method to correspond to the current intensity is feasible. However, it is important to note that the length of the conductor should be the same as the length of the grounding grid conductor to be measured next.

As observed from the figure, the induced electric potential, and the excitation current basically exhibited a positive relationship, which was consistent with the relevant theory. Therefore, we can use this relationship to correspond to the intensity of the excitation current by the induced electric potential in the measuring range.

**Figure 12:**
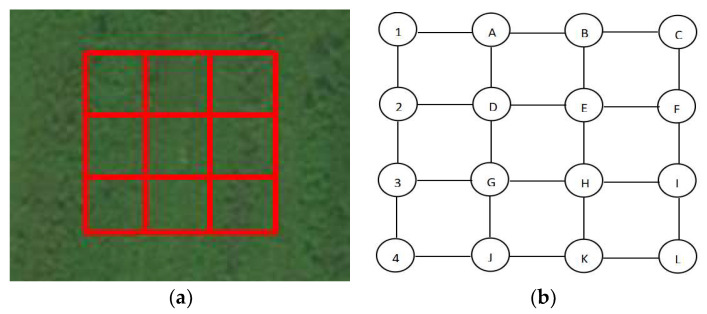
(**a**) The 3 × 3 grounding grid field laying diagram and (**b**) topology diagram of node labels.

Finally, we constructed a 3 × 3 “grounding network” in the more remote outskirts of Changchun, Jilin Province, China. A 3 × 3 “ground grid” means that each row and column has 3 square grids. As depicted in Figure 12a, the topology diagram is illustrated in Figure 12b. The lengths of the wires between each set of nodes are the same. The grounding conductor’s length between the grounding network nodes is generally ten meters or above. Therefore, we set the size of the grid lines to ten and fifteen meters for two different scenarios. We conducted experiments with conductors of different lengths and different resistance values, and the results are listed in Table 2.

As indicated in the table, we conducted three experiments for each group of conductors and obtained the average value for accuracy. We found that a higher conductor resistance produces a greater error value when the conductors are of equal length. We believe this is because the value of the current flowing through the conductor under test decreases under an increasing resistance, and the surrounding interferes more with the detection. Moreover, a greater distance between the nodes yielded a higher accuracy when the conductors exhibited, relatively, the same resistance. This is because a greater distance between the nodes poses less influence on the current in the other conductors’ effects for detection. Despite the errors, the proposed method achieved an overall accuracy of over 80%, demonstrating the feasibility of this method for detecting corrosion in grounding networks.

## 3. Conclusions and Discussion

In this section, we mainly summarize and discuss the scheme and results.

### 3.1. Discussion

Although the proposed solution is slightly more cumbersome than existing electromagnetic solutions, it offers maximum cost savings and further improves the electromagnetic method for corrosion detection in grounding networks, which is generally limited to the detection of breakage points. To avoid interference and the propagation characteristics of the alternating magnetic field, we selected a frequency of 380 Hz (low frequency). The strength of the current in the conductor under measurement was determined by the relationship between the induced electric potential and the excitation current. Note that the proposed method does not require the measured induced electric potential to be back-propagated step-by-step to solve for the strength of the induced current. The results of the field experiments demonstrate the feasibility of this scheme for applying low-frequency electromagnetic methods to detect corrosion in grounding networks, and the detection accuracy increased with the length of the conductors.

### 3.2. Conclusions

This research developed a specialized electromagnetic method for corrosion detection in grounding networks. Firstly, the conclude that this detection scheme, using a low-frequency electromagnetic method, is feasible through Comsol and Multisim simulation. We also concluded from simulation results and other references that grounding grid testing must be performed in dry soil, and the experiments in dry soil had essentially the same effect as experiments on the ground surface. Secondly, we proposed a new detection solution that overcame the general limitation of the existing electromagnetic methods used for detecting breakage points in the grounding network. Using a coil sensor, this solution measured the excitation current in the test conductor. The voltage value across the conductor could be measured using a voltmeter or other voltage detection device. The final calculation was performed considering R = U/I. Finally, we conducted relevant experiments on dry soil surfaces using suitable testing equipment, and the results proved the feasibility of this solution for the corrosion detection of earthing networks. Despite the convenience provided by the proposed method, a large amount of current must not be allowed to flow through the conductor under test or other grid conductors, as it could exceed the range and pose a safety hazard.

## Figures and Tables

**Table 1:** Various degrees of corrosion results.

Corrosion Level/Times	Multisim (mA)	Comsol (mA)	Difference Value (mA)
0	600.0	603.4	3.4
5	200.7	212.5	11.8
10	120.1	135.2	15.2

**Table 2:** Different degrees of corrosion results comparison table.

Port Number/m	Actual Resistance/Ω	Actual Test 1/Ω	Actual Test 2/Ω	Actual Test 3/Ω	Average/Ω	Error Value/Ω
GH/10 m	1.142	1.047	1.057	1.049	1.051	0.091
GH/10 m	6.139	5.217	5.214	5.223	5.218	0.921
GH/10 m	12.119	9.802	9.789	9.794	9.795	2.324
GH/15 m	1.168	1.077	1.092	1.089	1.086	0.082
GH/15 m	6.163	5.633	5.627	5.636	5.632	0.801
GH/15 m	12.141	10.069	10.079	10.083	10.077	2.064
